# M. F. Staniland

**Published:** 1939

**Authors:** 


### MEABURN FRANCIS STANILAND, M.R.C.S., L.R.C.P.

On 18th December, 1939, Dr. M. F. Staniland passed away after a long illness. He was the son of a Bristol doctor, and entered the Bristol Medical School in 1888.

Dr. Staniland who took the diplomas M.R.C.S. and L.R.C.P. in 1894, married Miss Leach, daughter of Dr. Leach, of Sturminster Newton, Dorset (Coroner for that area), who predeceased him.

He had practised in the Stapleton Road and his patients, no less than his colleagues, held him in high and affectionate esteem. His interest in the Bristol Medical School and its alumni never flagged and he was well known to many generations of students after his own time.

To his daughter, Mrs. Geoffrey Goodenough Taylor, we offer our deep sympathy.

